# Construction of patient trajectories to model clinical trial outcomes: application to myasthenia gravis

**DOI:** 10.3389/fdgth.2026.1755031

**Published:** 2026-04-22

**Authors:** Marc Garbey, Quentin Lesport, Henry J. Kaminski

**Affiliations:** 1 Care Constitution Corp, Houston, TX, United States; 2 LaSIE, UMR-CNRS 7356 University of La Rochelle, La Rochelle, France; 3 Department of Neurology & Rehabilitation Medicine, George Washington University, Washington, DC, United States

**Keywords:** clinical trial, clustering techniques, digital twin, muscular weakness, myasthenia gravis, neurology disease, precision medicine

## Abstract

**Introduction:**

Accurate prediction of patient outcomes in clinical trials is crucial for the timely assessment of treatment efficacy. This study proposes a novel approach to predict patient response using longitudinal clinical data.

**Methods:**

We construct temporal trajectories from longitudinal data and extrapolate these trajectories to forecast individual patient outcomes. Additionally, we assess when new patients align with established response patterns. The approach is evaluated using data from the MGTX trial involving patients with myasthenia gravis.

**Results:**

Our analysis demonstrates the predictability of patient trajectories and enables automatic clustering of patients based on treatment success. The clustering reveals potential associations with age and smoking status.

**Discussion:**

These findings highlight the potential of trajectory-based methods for early prediction of treatment response in clinical trials. We also discuss possible confounding factors that may influence the observed associations and predictive performance.

## 1 Introduction

Clinical trials are fundamental to evaluating new therapeutic interventions, yet they face numerous challenges, including patient heterogeneity, inconsistent data acquisition, and limitations in sample size. These issues are particularly pronounced in rare diseases, where small patient populations and variable disease trajectories complicate robust outcome predictions. In addition, the variation of pathophysiological mechanisms across subjects is often poorly defined or unknown. Mathematical modeling offers the potential to enhance clinical trial planning and more deeply understand trial results. The most advanced model of a human subject would be a digital twin. Originally conceived by NASA, digital twin technology has been broadly adopted across various industries and is now being applied to healthcare (1–4). Digital twins have demonstrated value in augmenting control arms in common disorders such as oncology disorders, in Alzheimer's disease, and in multiple sclerosis trials by reducing sample size requirements through synthetic control cohorts (5–7). In rare diseases, modeling would be particularly impactful to enable more efficient clinical trials by identifying early responders, predicting treatment efficacy, and optimizing trial design.

In this study, we propose a modeling framework aimed at enhancing the efficiency and predictive power of clinical trials, using myasthenia gravis (MG) as a representative example of a rare disease (8). MG is an autoimmune neuromuscular disorder caused by antibodies directed toward proteins on the postsynaptic surface of the neuromuscular junction. Symptoms range from disabling visual impairment, which typically occur at onset, to life-threatening respiratory failure. MG has a bimodal age distribution, with early-onset disease (typically <50 years) being more common in women, and then a male predominance after the age of 50. Patients suffer from a median delay from symptom onset to a diagnosis of approximately 12 months. Prevalence ranges from ∼15 to 25 per 100,000 globally and shows modest racial and ethnic variation, with higher reported incidence in Black populations in the US compared with White populations and propensity of restricted visual disturbance in some Asian populations. Despite recent advancements, there remains a significant unmet need in MG treatment because of poor adverse effect profiles of existing therapies, wide variation in patient response, and the fact that nearly a third of patients remain treatment resistant. To our advantage, MG serves as a good starting point for our work. (1) The fundamental pathogenesis of MG is well understood. (2) There have been several rigorously performed clinical trials over the past decade with potential access to data to allow model formation. (3) The specificity of the acetylcholine receptor (AChR) antibody testing provides confidence in patient diagnosis, which is critical for analyzing electronic medical record data. However, predictive modeling in MG faces challenges such as disease rarity, fluctuating manifestation over time, and the yet-to-be defined variation of autoimmune pathology among subjects.

We developed a predictive model for patient outcomes based on the concept of patient trajectory, or a time-based path tracking key clinical metrics, such as improved quantitative myasthenia gravis (QMG) and MG-Activities of Daily Living (ADL) scores, reduced prednisone dosage, or achievement of minimal manifestations (9–11). All these metrics are validated outcome measures and used to varying extents across clinical trials of MG. Constructing models for clinical trials presents several challenges: inconsistent timing of metric acquisition across patients, inherent uncertainty in neuromuscular disease scoring, and human factors affecting data accuracy. Given that MG trials typically enroll approximately 150, or fewer subjects, predictive modeling must balance the need for sufficient data diversity while ensuring that patient populations remain comparable.

To develop and validate our approach, we utilized data from the randomized, single-blind, phase 3 trial of prednisone plus thymectomy vs. prednisone alone (MGTX) trial, which demonstrated the added benefit of surgical removal of the thymus based on overall lower prednisone dose and QMG scores (9). The 3-year trial serves to exemplify the complexities of clinical trial design and optimization with respect to model building, which requires integrating numerous inputs—age, gender, disease duration, medical history, and diagnostic studies—to generate comprehensive disease trajectories.

This investigation presents an initial step toward constructing patient trajectory models, assessing their predictability, and exploring their potential for extrapolation in future clinical trials. We aim to provide insights into key control factors that impact modeling outcomes, laying the foundation for broader applications in clinical research beyond MG.

## 2 Materials and methods

### 2.1 Hardware

All computations were done on a laptop equipped with 11th Gen Intel® Core ™ i7—3 GHz and 16 GB RAM.

### 2.2 Dataset

A complete deidentified clinical dataset of the MGTX (NS 42685) trial was provided to the investigators by National Institutes of Neurological Disorders and Stroke) (5). This 3-year trial investigated the effectiveness of thymectomy plus prednisone vs. prednisone in patients with generalized, AChR antibody-positive MG between the ages of 18 and 65. During the course of the study, subjects were placed on a standardized dose of prednisone that was adjusted based on the change in quantitative MG score with dose escalation if the condition of the patient was worsening. For severe worsening as assessed by the site investigator, rescue therapies of intravenous immunoglobulin (IVIg) or plasma exchange could be instituted. After year 1, azathioprine therapy could be added for subjects with poor treatment response again as judged by the site investigator. One hundred twenty-six subjects were enrolled and followed up over a period of 36 months. The primary outcomes were the time-weighted averages of the QMG score and prednisone dose. The ADL and QMG scores were to be done from study baseline at month M0, M3, M4, M6, and then every 3 months to end of study.

#### 2.2.1 General approach

The concept of patient disease trajectory has evolved dramatically thanks to the use of big data (12) and artificial intelligence (13, 14). As we are dealing with a clinical trial of patients with MG, we acknowledge that our dataset is small in particular compared with similar work using the EHR data (12), and it would also be noisy due to the local temporal fluctuating manifestations typical of MG. Rather than using LLM and deep learning that requires large datasets and an accurate training dataset, our approach uses an unsupervised machine learning technique with a simplified mathematical framework formulation. Comparative approaches in clinical trials involve statistical techniques using Latent Class Analysis (LCA) and Profile Analysis (15) or Markov process methods (16). In our study, we require a 1) data structure of an object/process, and 2) a means of propagating that data structure through time to generate virtual trajectories (how a patient is doing).

For (1), we built a mathematical framework that defines the state variable and control variable of the patient trajectory. The state variable has patient outcome and clinical information along with patient history and comorbidities. The control variable is what the physician or a patient can impact, for example drug choice, drug dosage, surgery, diet, and likely other factors. (2) is described as a mathematical operator Φ*.* We project the subject's data from the clinical study into a mathematical object; by design, a projection provides a partial view and not an absolute reproduction of the real clinical patient condition. The projection must fulfill our goal of outcome prediction, such as improved QMG, MG-ADL, lowered dose of prednisone, or achievement of minimal manifestations, which are common trial metrics.

**Figure 1:**
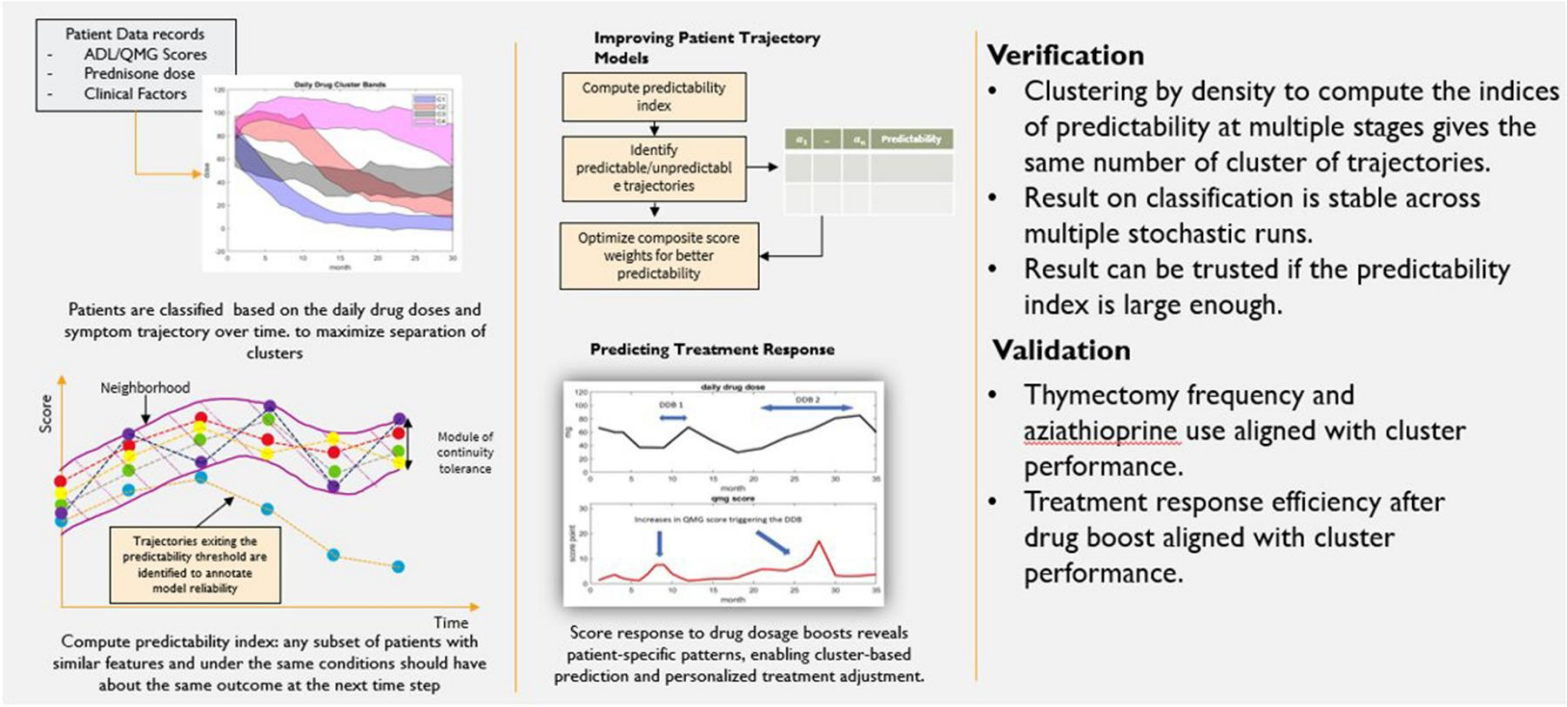
The model development process.

We developed a unified patient trajectory clustering technique that leverages data from several clinical trials to (a) compute an index of predictability for patient symptom evolution, and (b) quantify the impact of factors such as smoking, age, and BMI on drug dosing and patient outcomes. The method is summarized in Figure 1.

### 2.3 Patient trajectory technique

Let us denote $V_{0},V_{1},\ldots ,V_{q}$ the sequence of visits for a subject enrolled in a clinical trial, where *q* is the number of visits for data acquisition. Typically, the timing of visits varies to some extent for each subject. The dataset may require going through a few steps of preprocessing: Trials often have missing entries, for example due to scheduling problems or an incomplete time series because a subject leaves the trial. The time interval between two visits could vary among subjects. We use a linear interpolation technique to project the state variable on a uniform time interval series and discard a subject dataset when time gaps are too large to be filled with interpolation. The criterion is based on standard interpolation error estimates to stay within the observed noise level in the dataset. This error is proportional to the roughness of the signal, that is, the second-order derivative estimate. Most importantly in our situation, linear interpolation satisfies a maximum principle that does not amplify the effect of temporal rapid local fluctuation and damp noise.

Let ${\vec{X}}_{i}^{0},\;i=1,\ldots ,N$ be the vector of features or state variable for patient *i* at the initial time of the visit $\left(V_{0}\right)$.

Let ${\vec{X}}_{i}^{j},i=1,\ldots ,N,j=1,\ldots ,q$ be the vector of features or state variable for patient *i* at the time of visit $V_{1}\;$ to $V_{q}$, corresponding, respectively, to *j* = 1, …, *q*.

Thanks to the preprocessing step, the new vector feature is all set at the same time interval. Using the same notation ${\vec{X}}_{i}^{j},i=1,\ldots ,N,j=1,\ldots ,q$ for this new vector feature, we can draw the patient trajectory $P_{i}(t)$ as the set of state values that progress with time.

A further step in preprocessing the dataset might be denoising the time-dependent trajectory with a standard filtering technique: we assume that $P_{i}(t)$ is a continuous time-dependent function in the interval of time where no known singular events such as hospitalization occurred. Hospitalization may correspond to the step value in the trajectory.

One can cluster the dataset of trajectories $\;P_{i}(t)$ on a given time interval of continuous variation in order to check whether patients may share similar behaviors. To improve our approach from our previous work (17), instead of K-means that classify the trajectory dataset point wise, we use K-Medoids (PAM) combined with Dynamic Time Warping (DTW). DTW “stretches” or “compresses” the time axis to find the best alignment between two trajectories (18). If two patients have the same “overshoot” pattern but are slightly shifted in time, DTW will recognize them as similar. K-Medoids picks an actual patient trajectory as the center of the cluster (the medoid), rather than a calculated “average” (the centroid). This makes the result much more interpretable and less prone to being pulled away by outliers. This algorithm is readily available as a matlab function of the signal processing tool box (https://www.mathworks.com/help/signal/ref/dtw.html).

The quality of the clustering depends on the separation of the clusters: such indices of separation are the Davies–Boulin Index (6), the Calinsky–Harbasz Index (7), or possibly the Dunn Index (19). All these indices use some variation of the silhouette function defined as follows (20). We use the DTW distance for the computation of the distance between patient trajectories.

Validation of the clustering technique is provided by visualizing the silhouette portrait as in (20) but also looking at the result that is the average trajectory for each cluster and its standard deviation across patients for that cluster. We expected standard deviations along the trajectories that show clearly non-overlapping average trajectories (in space-time).

### 2.4 Patient trajectory predictability

The clustering of patient trajectories relies on the assumption that disease progression is not random and can be predicted along a specific trajectory. However, predictability is not guaranteed, as latent variables omitted from the dataset can introduce significant noise. Building a predictive model from data that do not support it can lead to spurious conclusions. Consequently, we transition from static clustering to a dynamic stability analysis, bridging the gap toward an effective dynamical system approach (18, 21) suitable for small, noisy clinical trial datasets.

To quantify this, we define a predictability index. If a dataset supports predictability, the operator Φ must exhibit a continuity property: any subset of patients with similar baseline features under identical conditions should yield comparable outcomes at the subsequent time step. This is formulated as follows:

$$\left|\Phi (x_{1})-\Phi (x_{2})|\leq K|x_{1}-x_{2}\right|$$

where *K* represents the modulus of continuity. For a reliable prediction based on (x,input), we target K≈1. This evaluation is applicable to both continuous variables, such as the drug dosage (the primary outcome of the MGTX trial), and discrete variables like the QMG score or the MG-ADL score.

To enhance the robustness of this index against the local time fluctuations typical of neuromuscular diseases, we implement two strategies:

- Temporal Blocking**:** Rather than calculating distances at a single time step $j$, we use blocks of successive visits $\left[e.g.,\;\mathrm{from}\;(t_{j},\ldots ,t_{\;j+k})\;\mathrm{to}\;(t_{\;j+1},\ldots ,t_{\;j+k+1})\right]$. This extends the norm calculation across both space and time to stabilize the predictive model.
- Neighborhood Stability**:** Instead of tracking single-patient continuity, which is highly sensitive to noise, we monitor whether groups of patients in the same neighborhood remain together at the next interval.

To implement, we employ a clustering method that partitions the multidimensional space into non-empty spheres of diameter *D*, similar to the approach in (22). We utilize a Greedy Covering Approach that prioritizes high-density regions while identifying singular outliers.

By allowing spheres to overlap, we perform a maximal covering location problem (MCLP) (18). This is crucial for clinical data, where patients rarely fall into mutually exclusive silos. The algorithm iteratively selects the most populous spheres first to ensure that the predictability index is built on the strongest statistical evidence. Patients in spheres falling below a minimum occupancy threshold are treated as “singular,” suggesting that their states are too rare for group-based prediction.

### 2.5 Algorithm and predictability index $I_{p}$

Let the partition of the dataset be $\Omega =\cup \Omega_{k}$, where each subset has a diameter ≈D. The algorithm proceeds as follows:

1. For each trajectory $x_{i},$ calculate the number of neighbors within distance D.
2. Identify the sphere centered on $x_{i}$ with the largest number of elements $N_{i}.$
3. Remove these trajectories from the dataset and repeat the process until all patients are exhausted or categorized as singular.

Finally, we test the continuity of the operator Φ by checking how many trajectories from an initial sphere $\Omega_{k}^{j}$ remain within a predicted sphere of diameter *D* at the next time interval. The predictability index $\;I_{p}$ is defined as the percentage of patients who remain within their predicted sphere. An $I_{p}$ of 100% indicates a continuity modulus of one, while a lower $\;I_{p}$ suggests that mean-trajectory extrapolation is less reliable.

### 2.6 Remarks on how to improve the trajectory model

This index of predictability can be used to select the best dataset to build a predictive model. One can formulate this as an optimization problem that can be achieved by one of the standard stochastic optimization algorithms, such as the genetic algorithm (23), which is a method for finding solutions for constrained and unconstrained optimization problems that are based on natural selection. Alternatively, the dataset of subjects can be partitioned into two categories: those in whom we can use extrapolation of trajectories and those that do not fit. From this partition, we can mine data on the control variable subset $\left\{{\vec{Y}}_{i}\right\}$ and corresponding patient data $\left\{{\vec{X}}_{i}^{j}\right\}$ to extract some statistics of the features that may provide an explanation on the lack of predictability of these subject trajectories.

We may find that the combination of both techniques is best to optimize selection of variables that drive predictability of the model.

Another option that we have tested is to focus on the prediction of a score and adjust the weight of each feature of that score to increase the quality of the prediction, that is, maximize the predictability index. The ADL score, for example, is assembled from the individual score based on separated evaluation of muscle groups. The standard ADL score takes the sum of 8 such metrics, but there is no specific rationale to say that the ptosis score should be as significant as the sit-to-stand score. With our methodology, we look for the optimum weight combination of these 8 individual scores that best predict the patient’s condition at the next time interval. Let α be the unknown 8 components vector and $\mathrm{AD}L_{j},j=1,\ldots ,8$ the individual score in (0, 3) for each muscle group.

The optimization problem can be written as follows:

$$\underset{\sum_{\;j=1}^{8}\alpha_{j}\mathrm{AD}L_{j}}{\max}\;\{I_{p}\},\;\mathrm{under}\;\mathrm{the}\;\mathrm{constraint}\;\sum_{\;j=1}^{8}\alpha_{j}=8,\;\alpha_{j}>0$$

In our model, the weight vector should be independent of the patient and time of the visit indeed. We will define this modified ADL score: $\sum_{\;j=1}^{8}\alpha_{j}\mathrm{AD}L_{j}$ as a new ADL composite score. Following the same idea, we can combine the ADL score and QMG score into a weighted combination that maximizes the predictability index. Once again, the finding of the optimum weight factors can be formulated as an optimization problem:

$$\underset{\gamma \cdot \mathrm{ADL}+(2-\gamma )\cdot \mathrm{QMG}}{\max}\;\{I_{p}\},\;\mathrm{under}\;\mathrm{the}\;\mathrm{constraint}\;\gamma \in [0,2]\;$$

However, one technical difficulty is related to the use of the control variable ${\vec{Y}}_{j}$. Subjects may vary in sensitivity to a treatment with the same exact condition $\vec{X}$. Some subjects might be highly responsive to a small drug dosage, while others require a larger dose. A possibility to get rid of that scaling factor would be to normalize ${\vec{Y}}_{j}$ for each patient, within the same cluster, using the total amount of drug or thymectomy status. We emphasize this point as it is unquestionably the case for corticosteroid responsiveness and thymectomy response.

### 2.7 Toward a model to generate patient trajectories

We will now introduce a new concept to construct a simplified, first-principles mathematical model to predict patient score trajectories from daily drug administration. This model is intended to serve as the foundation for a readily implementable tool that optimizes drug dosage according to patient-specific disease severity, thereby enhancing disease control.

The MGTX protocol for prednisone dosing was decided *a priori* by a consensus decision of experts and was as follows: Participants who were not already receiving prednisone at study entry received an alternate-day dose of oral prednisone starting at 10 mg and increased in 10 mg steps to 100 mg on alternate days or to 1.5 mg per kilogram of bodyweight, whichever was lower. For participants who were already taking prednisone, the dose could be increased up to 120 mg in those who did not reach minimal-manifestation status by month 4. Minimal-manifestation status (MMS) is defined as “no symptoms or functional limitations from MG, but there may be some weakness on examination of some muscles” (24).

At month 4, the prednisone dose was maintained until minimal-manifestation status was reached and the QMG score was less than 14 and had also fallen at least 1 point below baseline, as determined by an evaluator who was blinded to the trial-group assignment. The alternate-day prednisone dose was then reduced by 10 mg every 2 weeks until a level of 40 mg was reached, with subsequent slowing of the taper to 5 mg every month, as long as MMS was maintained. If MMS was lost, the alternate-day prednisone dose was increased by 10 mg every 2 weeks until the status was restored. Tapering could resume 4 weeks later.

Once prednisone tapering commenced, the total dose of pyridostigmine, a cholinesterase inhibitor that produces rapid but transient improvement in strength, could not exceed 240 mg per day. Plasmapheresis or intravenous immune globulin was permitted at the discretion of the unblinded neurologist in patients whose condition was unstable, but it was not permitted to maintain MMS. Patients who did not have MMS at 12 months or who had an unacceptable level of side effects with prednisone could receive azathioprine at a dose of 2.5 mg per kilogram per day or another immunosuppressant such as cyclosporine if azathioprine caused unacceptable side effects.

To model patient trajectories, we must quantify the patient's score response to drug dosage increases. The existing protocol employs a function *Y*(*S*,*t*), where *S* represents the score and *Y* the daily drug dose, which is set *a priori* the same for all patients. To develop patient-specific dosage regimens, rather than relying on generalized guidelines, we analyze the score response to isolated drug dosage boosts (DDBs). Ideally, the score should decrease following a DDB, although this is not always observed. Utilizing the MGTX dataset, we extract 78 DDBs and corresponding score responses to identify patterns in this relationship.

**Figure 2:**
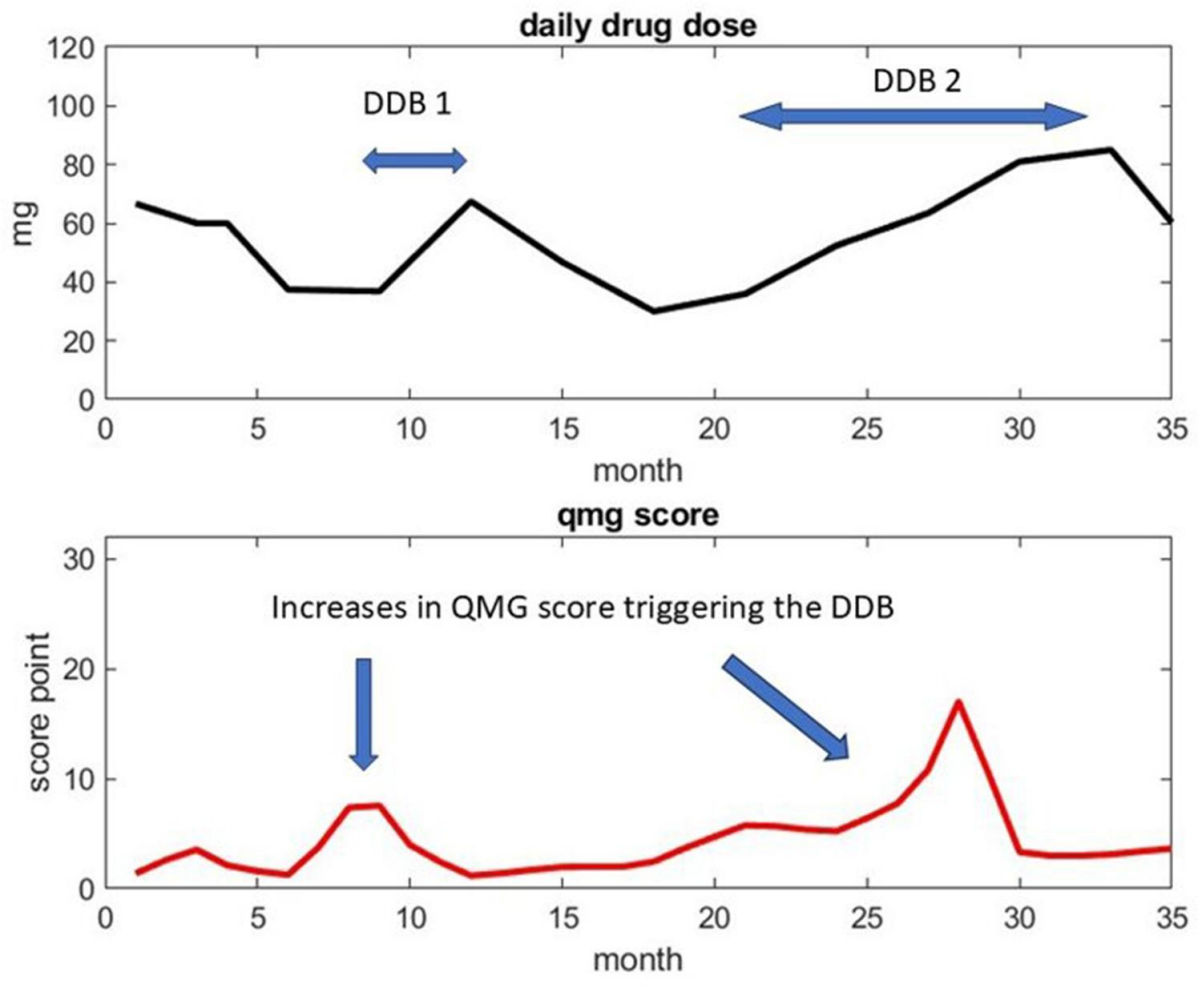
An example of daily drug dosage and drug response with one patient from cluster 3. The first DDB occurs at month 10 as a result of the sudden increases of the QMG score at month 9. The second DDB occurs at month 21 and represents a continuous but moderate increase of daily drug dosage. One appreciates that the decision of the clinician on increasing drug dosage from QMG score observation reflects their own appreciation on the risk of drug increases.

We hypothesize that these patterns are correlated with disease severity, specifically the patient clusters previously identified. Figure 2 illustrates the concept, showing daily drug dosage and score fluctuations for a patient in cluster 3. While a DDB should ideally be followed by a score decrease in responsive patients, clinical practice introduces variability, as clinicians adjust dosages based on experience. Nonetheless, we obtain multiple dynamic response samples from longitudinal datasets, which serve as the fundamental units for constructing cluster-specific response maps.

**Figure 3:**
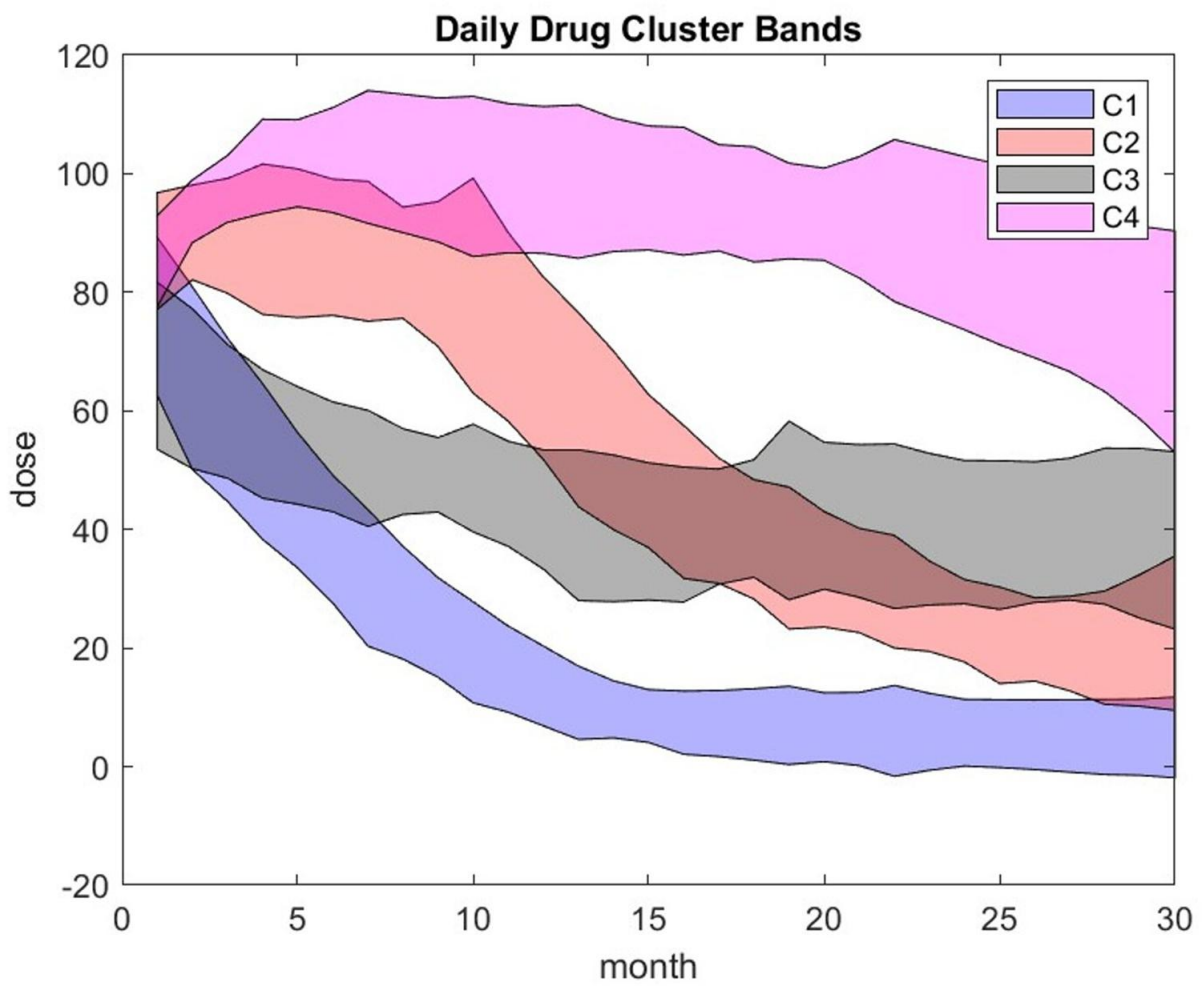
Patient trajectory over time representing the daily prednisone dose corresponding to the 4-cluster result.

Given a sufficiently large database of dynamic responses per cluster, we can create a predictive map of drug dosage strategies that aim to control the score without relying on universal protocols. To assign a new patient to a cluster, we first compare their initial trajectory to reference trajectories (Figure 3). Subsequently, we verify that the patient's DDB-to-score-change (*Δ*S) response patterns align with the cluster's DDB database throughout treatment. This approach forms the basis for a novel machine learning algorithm for personalized drug delivery management.

Next, we describe the main results obtained with our novel methodology.

## 3 Results

### 3.1 Clustering analysis based on daily prednisone and patient score

The distribution of the MGTX patient population with respect to age and BMI is shown in Supplementary Figure A1 of the appendix. Seventy-one percent of subjects are women, consistent with the demographics of MG given the age distribution of the trial. Out of 126 subjects, 111 patients have a record available covering 140 weeks. We exclude from the analysis the first 10 weeks, which was the time period of required dose adjustments, and the unlikelihood that an emerging pattern could be observed. We start the analysis by clustering the trajectories of the daily prednisone dose. The strips are centered on the mean trajectory of bandwidth ± one standard deviation for each 4 clusters as shown in Figure 3. We order the cluster tags from 1 to 4, to correspond to the best to worst performance outcome. The numerical result of the clustering is stable, that is, it gives the same computational result for each run.

**Figure 4:**
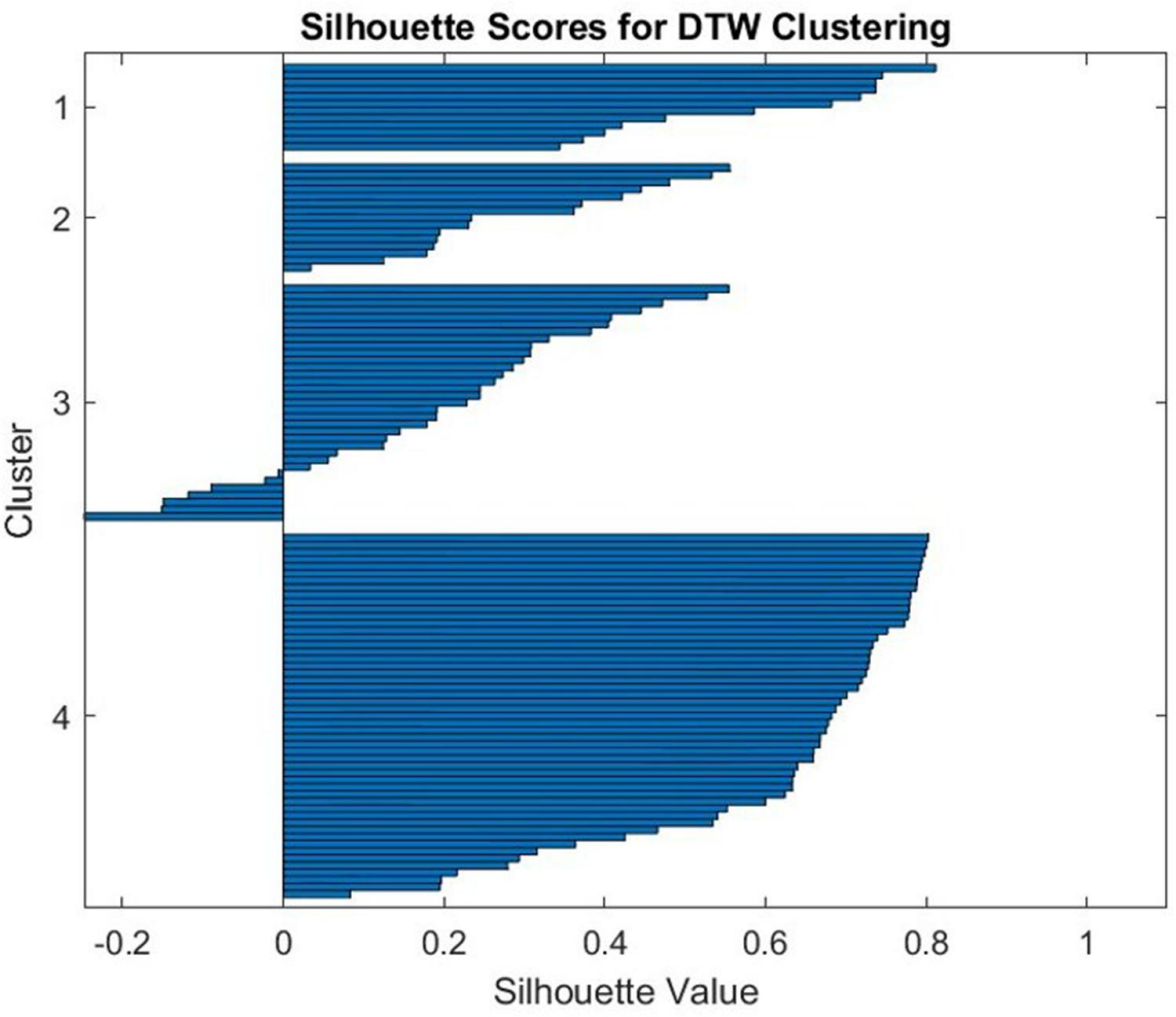
Silhouette value for the 4 clusters based on daily prednisone dose; the MGTX trajectory was computed with 111 patients that had a complete record for the 36-month period. Five patients out of 111 had a negative silhouette, which means they are not properly classified. They were removed from the analysis of the impact of age, sex, smoking, and BMI.

The maximum number of clusters that provide a good separation between clusters is 4: out of 111 patients, five have significant negative silhouette function and two are border line—see Figure 4. The main 4 outliers – most negative silhouette – seems to correspond to patients with drug prescriptions that differ substantially from the protocol; see Supplementary Figure A5.

We compute the index of predictability on daily prednisone dose using a partition with subsets of diameter of the order of 8% of the maximum dose. This diameter guarantees that there are enough spheres with 10 patients or more to classify the trajectories. The algorithm shows that the number of spheres in the partitioning varies from 3 to 4 with the time interval of prediction, which is coherent with the previous clustering algorithm result.

The index of predictability for the primary outcome of MGTX is close to 90% and confirms the validity of using patient dose trajectory as a model. We removed the seven patients with negative signature from further analysis.

Almost half of the patients belong to cluster $C_{1}$ and have the best daily prednisone dose outcome. The pattern of the other three cluster trajectories is clearly different and well differentiated. From this result, we can search for the features that some of the patients in each cluster have the most in common.

**Figure 5:**
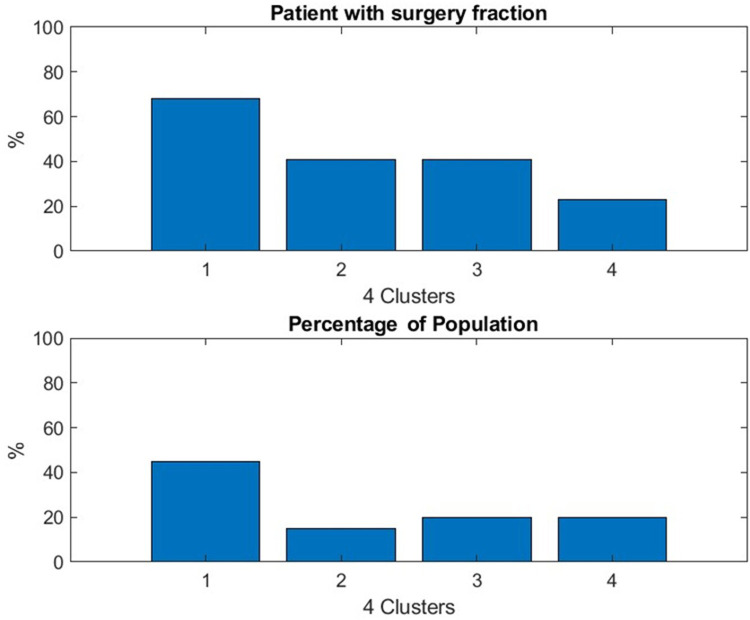
The result confirms the benefit of thymectomy for patient outcome expressed as daily prednisone dose. Patients with thymectomy were more common in Cluster 1, which identified the subjects who responded best to treatment.

**Figure 6:**
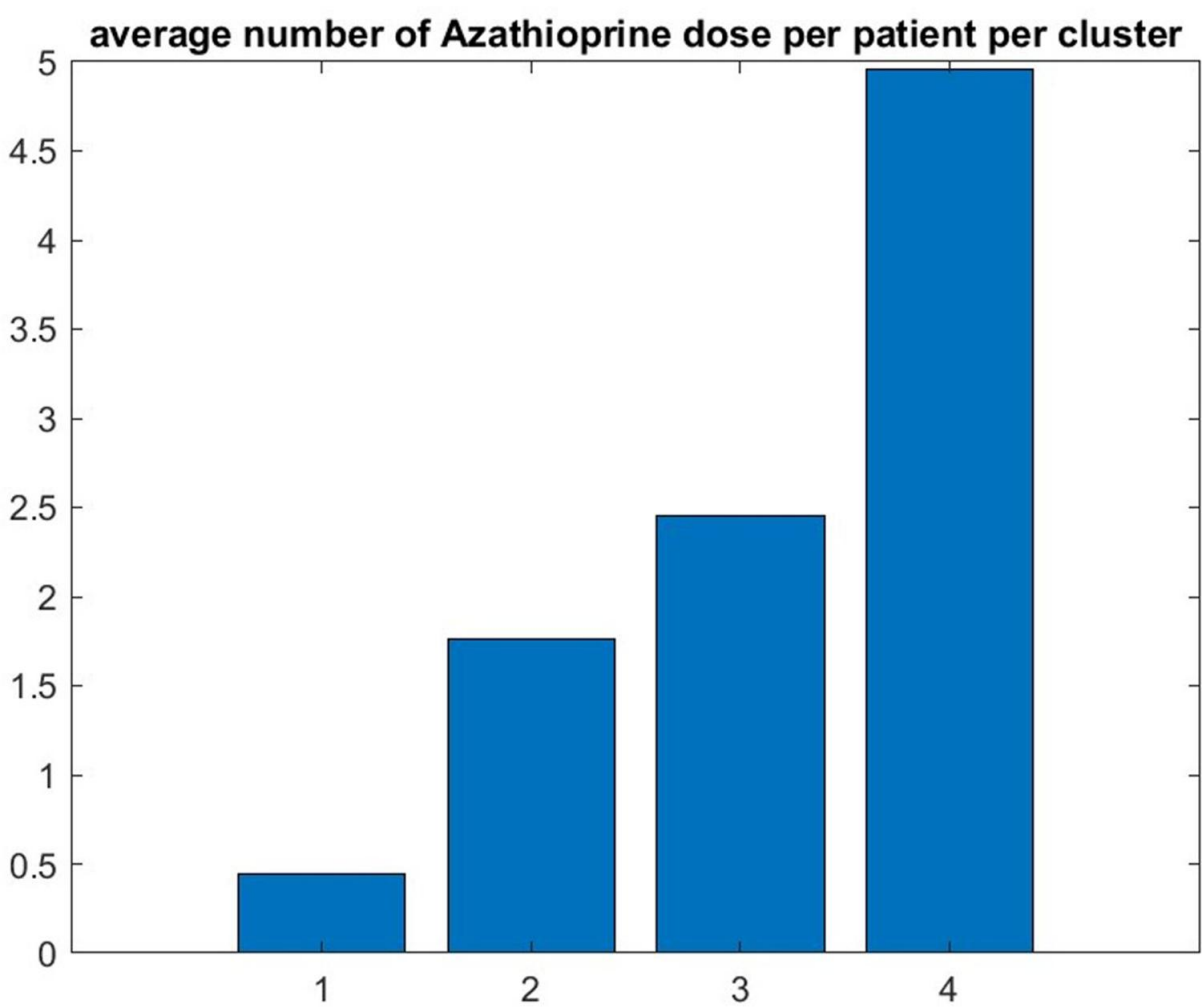
The graphic confirms that the patients who do best (respectively worse) have the lowest (respectively highest) number of doses of azathioprine.

First, we find that thymectomy is beneficial, consistent with the primary outcome of the MGTX trial in (5): the percentage of the patients who went through thymectomy decreases with the performance of the cluster—see Figure 5. As expected, the average number of doses of azathioprine, a metric of poor response to treatment, received by the patient increased as the cluster performance decreased—see Figure 6. Both results provide an indirect validation of the clustering result.

**Figure 7:**
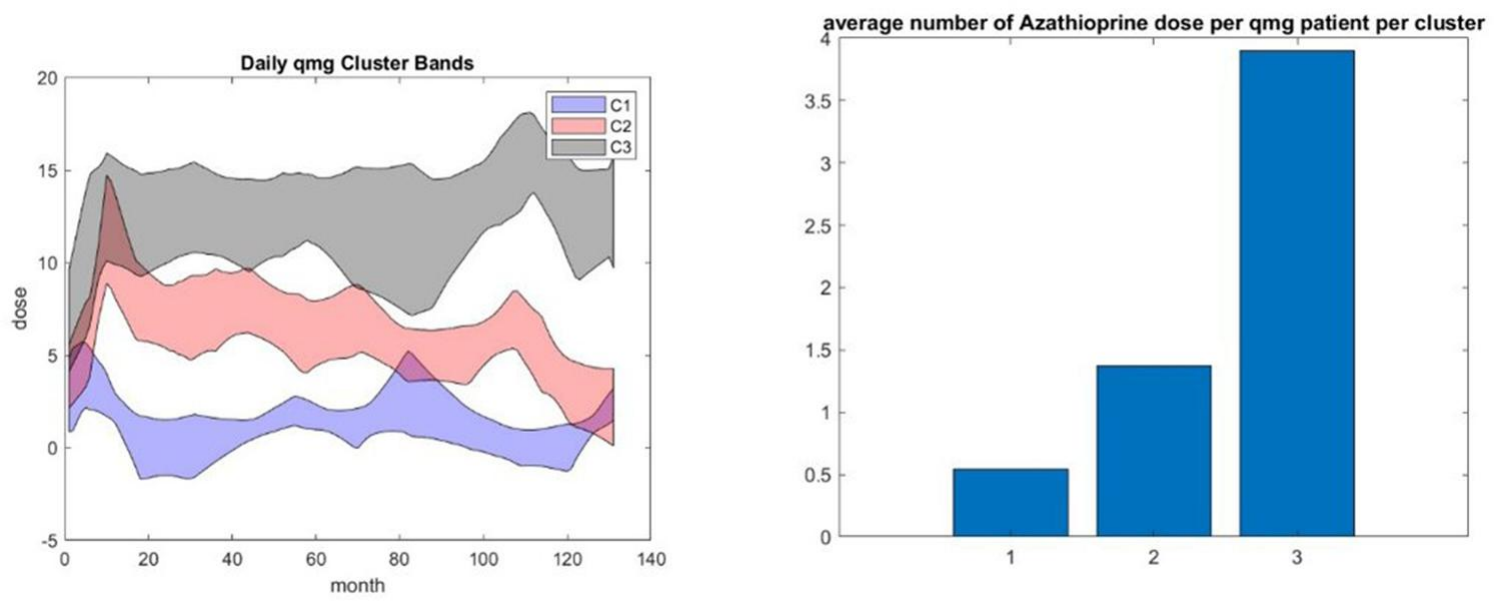
(Left) Patient trajectory clusters of QMG—(right) the corresponding average of azathioprine dose.

Clustering the patient trajectories for the QMG score gives similar results—see Figure 7 left and right, except that three clusters provide a better separation than 4. We assess whether smoking, BMI, gender, and age play a role in cluster performance given their established risk factors for autoimmune diseases. We obtain the most clear picture related to the influence of age, BMI, and smoking from the three clustering results of QMG and ADL patient score trajectories.

**Figure 8:**
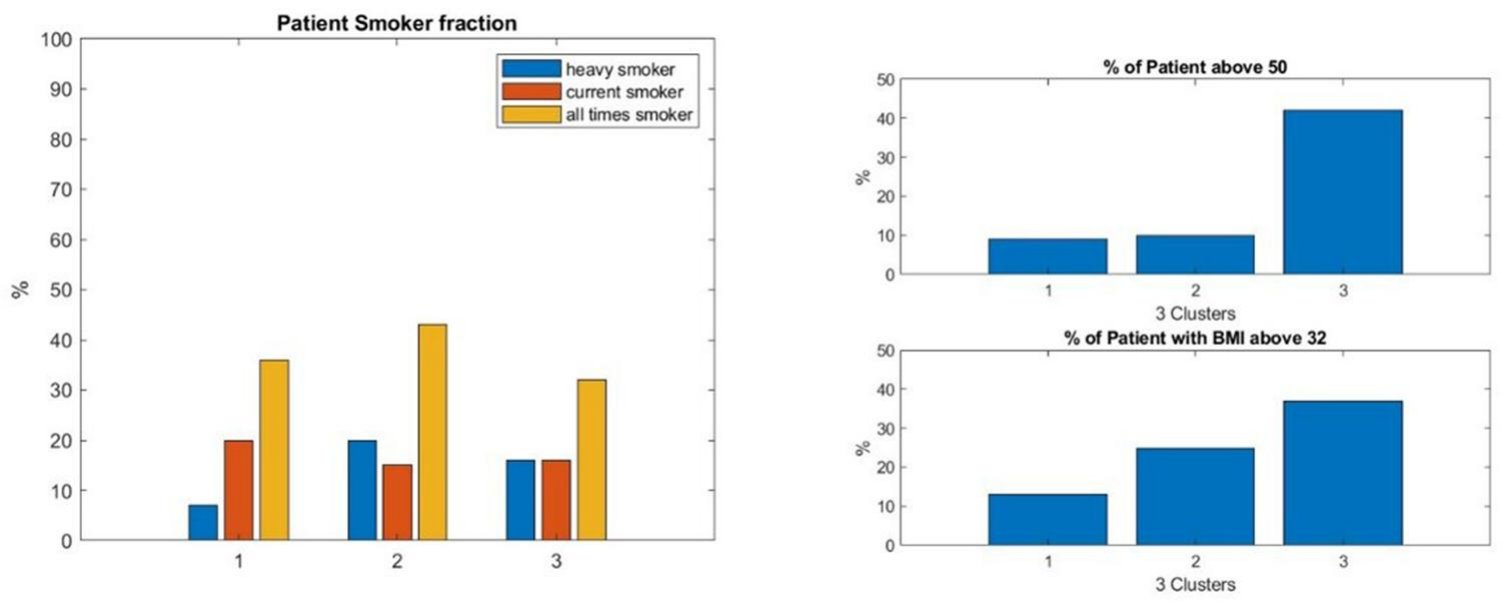
Both types of clustering—QMG and ADL patient trajectory clustering—show that there is a slight advantage to do not smoke for the efficiency of the drug treatment—see left. Both show that young age and low BMI provide a clear benefit—see right. This graphic is for the ADL trajectory clustering.

We found that smoking had a moderate negative impact; see Figure 8.

The average BMI of the population of each cluster decreases with the performance of the cluster. This cannot be the indirect effect of a relationship between age and BMI. We assessed that the average BMI of the MGTX patient below 50 and above 50 was not statistically different, but the distribution of these features was not a normal distribution as shown in Supplementary Figure A1.

We found a temporal pattern of the dose of azathioprine during the MGTX trial. Once a subject was on this drug, they typically maintained the drug. We can then define the time of failure of treatment as the time the patient starts azathioprine. Supplementary Figure A2 shows this pattern where each row represents a patient. The patients have been ordered from late failure at the bottom of the left graph to early failure at the top. The 70 patients who never received the drug are not shown. The right graph plots the accumulated dose of prednisone received by patients at the time of receiving azathioprine. There appears to be a saturation effect on the accumulated dose of prednisone that corresponds to the time of failure of the treatment—see the horizontal red line in Supplementary Figure A2 right.

**Figure 9:**
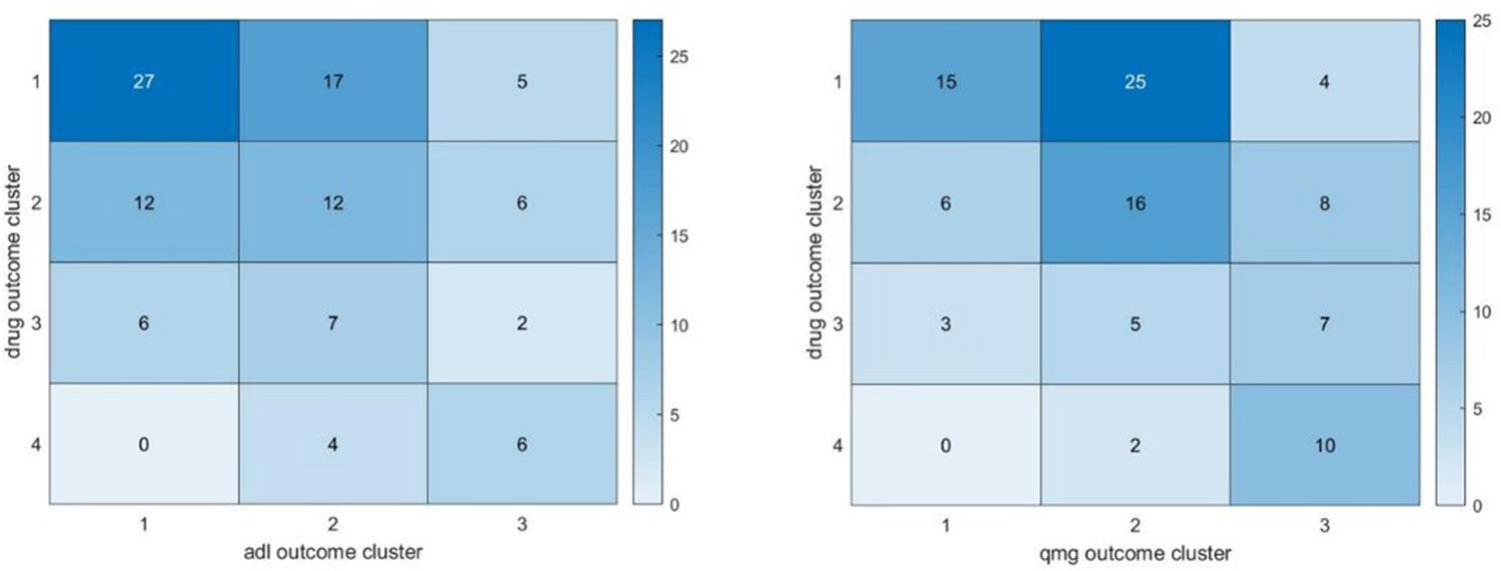
To compare the MGTX patient distribution between clusters for the daily prednisone dose, and the ADL composite score, we use a 3-cluster algorithm for both outcomes. This diagram sums up the results for 97/108 patients. Five patients (respectively 11) had a negative signature in the drug (respectively, composite ADL) clustering. The 3 by 3 heat map shows the distribution of patients in the intersection of the cluster. The box first row, first column corresponds to the number of patients who belong to the first cluster for the drug dose and to the first cluster for the ADL score. The box first raw, second column corresponds to the number of patients who belong to the first cluster for the drug dose and to the second cluster for the ADL score, and so on. On the right, we show a similar result that compares the MGTX patient distribution between clusters for the daily prednisone dose, and the QMG composite score.

The heat map of Figure 9 gives the distribution of patients at the intersections of the daily drug clusters and score clusters with ADL—left panel, respectively, QMG—right panel. These diagrams illustrate the results for 104 patients (respectively 101) patients out of 111, as we want to keep only those patients with a positive silhouette function for both metrics. As expected, the number of patients who belong to the best cluster for both daily drug and composite ADL outcomes is the largest. However, even for the best drug trajectory cluster (see first row of the heat map of Figure 9) one can observe that some patients' ADL scores are poorly controlled. We suspected that these patients would have benefited from a larger daily dose of prednisone or additional therapy. All patients outside the diagonal would correspond to a suboptimal control of the drug dosage that was defined *a priori* for all subjects in the MGTX protocol. The QMG score matching table (Figure 9, right panel) reinforces that concern mainly in patients belonging to Cluster 1 of drug prescription patient trajectories.

### 3.2 Improving the MG-ADL score formula—proof of concept

The ADL has eight individual categories to its score, while the QMG has 13. We found initially that the clustering on individual ADL or QMG score items such as eye lid drop, double vision, or arm weakness usually failed, with either the clusters not being well separated or having chaotic temporal behavior. Accordingly, the index of predictability of the ADL score was low and rarely reached 50% over time. By optimizing the weighted combination of ADL individual test scores, we were able to improve that index of predictability. The optimum weighted composition (see Supplementary Figure A3) provided a 70% average performance measured with the $L_{2}$ norm. It should be noted that we did a relatively coarse search on the optimum weight. The problem has 8 unknowns and one constraint: the overall sum of coefficients stays fixed. Nevertheless, the clustering of trajectory for the new weighted ADL score improves the separation of the three clusters (see Supplementary Figure A6)—which, in turn, may offer a better alternative to the standard ADL score formula with equal weight to predict trajectories once a patient is identified to belong to a cluster.

### 3.3 Non-linear dynamic of a DDB and score coupling with MGTX

**Figure 10:**
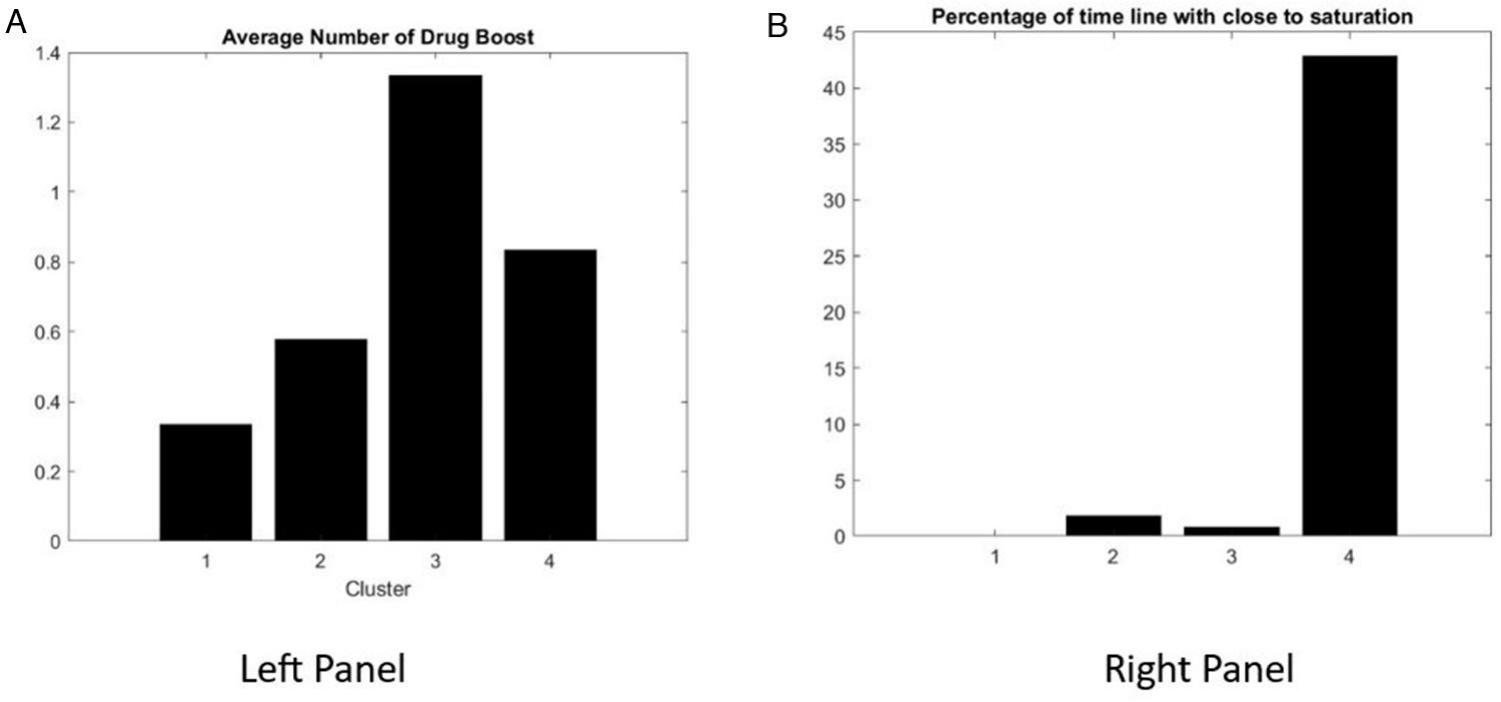
The average number of DDBs of patient daily drug per cluster **(A)** and the corresponding percentage of the time line during the clinical trial when drug dosage is close to its maximum allowed limit **(B)**.

To test the response of the patient score to a DDB, we computed the number of boosts of the drug prescription (see Figure 10 left panel) and the percentage of the timeline where the drug prescription is close to the maximum allowed limit (Figure 10 right panel).

The average number of DDB per patient per cluster is as expected low for cluster 1 (Figure 10, left). The results for the DDB characteristics corresponding to cluster 4 are particularly striking. The drug dosage is close to saturation and the opportunity to increase the drug dosage is limited. This result is in alignment with the performance of the patient illustrated by the primary outcome of the MGTX clinical study, that is, drug dosage. However, it shows that the protocol for patients in clusters 1–3 could have allowed a larger drug dose, and failure of treatment for the patient in cluster 4 could have been determined quickly.

## 4 Discussion

The present study introduces a novel framework for clinical trial outcome prediction by leveraging patient trajectories—a method that transforms longitudinal clinical data into dynamic, individualized response maps. By integrating a unified clustering approach with a rigorously defined predictability index, our methodology not only distinguishes differences in treatment responses among patients with MG within a clinical trial but also lays the groundwork for constructing digital twins. This innovative strategy captures the complex, time-dependent evolution of patient metrics, specifically, QMG, MG-ADL scores, and daily prednisone dosages, and provides a quantifiable means to assess treatment efficacy. The mathematical modeling, dynamic clustering, and personalized response mapping offer a powerful alternative to traditional, static approaches, which could be applied to the design and analysis of clinical trials, most importantly in rare diseases.

Our approach demonstrated that patient outcomes could be effectively stratified into distinct clusters that correlate with key clinical features such as age, smoking history, and thymectomy status. These clusters not only mirror known clinical relationships—for instance, the beneficial impact of thymectomy—but also reveal nuanced patterns of drug dosage response that traditional methods overlook, the variation in time of response to prednisone escalation among subjects, which has not been evaluated in clinical trials of patients with MG. The establishment of a predictability index further enables the evaluation of treatment consistency over time, addressing one of the critical challenges in clinical trial modeling: the variability inherent in small, heterogeneous patient populations.

Despite these promising findings, the study acknowledges several limitations. The model's predictive utility is constrained by the inherent noise of clinical data, including inconsistencies in data acquisition and protocol-driven variability in treatment adjustments; in particular, a clinical protocol may not be followed rigorously, and there is a known high variability and inconsistency of clinical scoring throughout the tests (25, 26). The drug dosage protocol relied on patient scores, acknowledging the moderate correlation between QMG and MG-ADL (27–29). The adoption of Minimal Manifestation Status reflects the field's recognition that optimal patient status does not necessarily equate to zero QMG and MG-ADL scores. Notably, MG-ADL exhibited a “floor effect,” limiting its ability to differentiate between patients with low disease severity, particularly within a clinical trial context. For example, patients with minimal clinical findings (e.g., slight weakness on arm extension) may exhibit normal functional capacity, leading to discrepancies between QMG and perceived wellbeing. However, we suggest that there are ways to improve the accuracy of clinical scores using video acquisition and artificial intelligence (26, 30–33). There is inherent noise within the dataset, which limits the achievable precision of probabilistic estimates for factors, such as age, BMI, and smoking, on drug responsiveness.

The concept of trajectory may inspire several techniques to proceed with modeling. It is relatively straightforward to model the response of the score to the drug dosage using a simple logistic model or sigmoid curve: see Supplementary Figure A5:

$$\dot{S}=\underset{\left(1\right)}{\underbrace{\beta \frac{Y}{Y_{\max}}S}}\;\;\;+\underset{\left(2\right)}{\underbrace{\;\;\alpha S\cdot (S_{\mathrm{capacity}}-S)}}$$

(1) and (2) may operate at different timescales: *α* is the time scaling factor that drives how fast the score may deteriorate without a drug. β is the time scaling factor that drives the delay to observe the drug effect on the score.

The potential equilibrium and endpoint of the sigmoid equation is

$$S=S_{\mathrm{capacity}}-\;\beta \;\frac{Y}{Y_{\max}}\;$$

The sigmoid curve is by no means the representation of the response of the autoimmune system to the daily drug. As a matter of fact, all assumptions on the behavior of the autoimmune response, neuromuscular transmission defect, and biological effect of drug sum up to the decision tree algorithm that defines the function *Y*(*S*,*t*), which is an observation of what the treatment protocol does for the patient. The sigmoid curve is simply a surrogate that enforces the saturation effect of drug and factor is an indicator of the disease severity expressed as $S_{\mathrm{capacity}}$.

We observed that this phenomenological model exhibits limited predictive utility because of several factors. First, the immune system response is inherently complex and subject to change over the nearly 3-year duration of the treatment trial. Second, the model lacks true predictive power without a more nuanced understanding of how drug dosage boosts (DDBs), administered in response to sudden disease worsening reflected by QMG and ADL scores, increase.

To address this, we shifted our focus toward a localized analysis, isolating DDBs for each patient and quantifying the corresponding score responses from the dataset. We hypothesize that a sufficiently large database of these localized response maps will provide the fundamental building blocks (“basic atoms”) necessary to reconstruct individual patient responses to daily drug dosage throughout the treatment period.

While we have neither achieved a comprehensive model construction, because of data limitations, nor have we optimized score input by identifying the optimal combination of MG-ADL and QMG scores for treatment guidance, we remain optimistic that the aggregation of data from multiple clinical trials will enable the development of a robust predictive model.

Although our modeling does not purport to reflect the autoimmune pathology of MG, we do think that there is the potential to reveal potential biological insights. Modeling of normal and autoimmune pathology is an active line of investigation (34–36). In this study, we noticed a distinct pattern of treatment response in a cohort of patients. Although variations in clinical response to corticosteroids are well appreciated for MG (37) and other autoimmune and inflammatory diseases (38), we found in this study that higher doses overcame some level of poor response, while some patients reached a plateau of improvement regardless of drug dose. This suggests that drug metabolism relates to treatment response, which is consistent with the findings of a metabolomic analysis of MGTX subjects that indicated xenobiotic pathways were related to poor outcomes (39).

## 5 Conclusion

This study paves the way for a paradigm shift in clinical trial design for MG and similar conditions. By capturing the dynamic interplay between patient characteristics and treatment responses, our trajectory-based approach not only enhances the precision of outcome predictions but also informs the development of personalized therapeutic strategies.

Furthermore, while our trajectory-based clustering provides an innovative lens through which to view patient responses, its full potential hinges on the availability of larger, more diverse datasets that can refine the model's parameters and enhance its generalizability. As data integration and computational power continue to evolve, this method holds significant promise for improving patient care and advancing precision medicine in the realm of MG but can easily be generalized to many other disorders. Future work will focus on integrating additional data sources—such as real-time digital biomarkers, video-based assessments, and electronic medical records—to further validate and optimize our modeling approach to lead to a digital twin.

## Data Availability

The data analyzed in this study are subject to the following licenses/restrictions: The data analyzed in this study were obtained from the National Institute of Neurological Disorders and Stroke (NINDS) through approved data-use requests for the MGTX clinical trial. Due to participant privacy and data-use restrictions, the dataset is not publicly available. Requests to access these datasets should be directed to NINDS at https://www.ninds.nih.gov.
