# Supplementary material for: Construction of patient trajectories to model clinical trial outcomes: application to myasthenia gravis
Source: Front Digit Health. 2026 Apr 22;8:1755031. doi: 10.3389/fdgth.2026.1755031 (PMC13143922; doi:10.3389/fdgth.2026.1755031)
Supplement: Supplementary file 1 [file Datasheet1.docx]

Supplementary Material

# Supplementary Figures and Tables


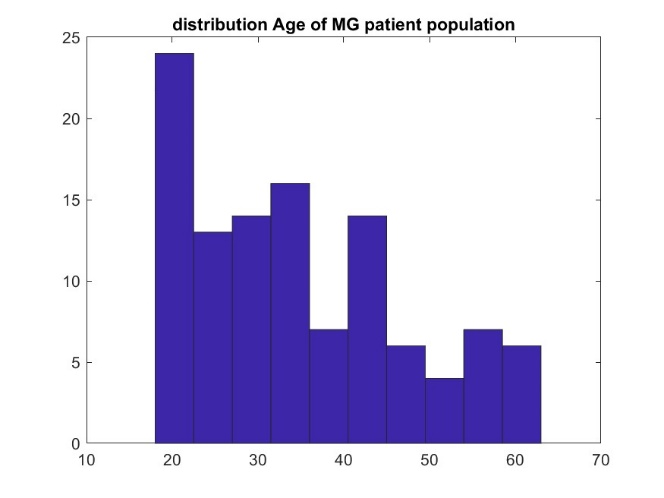

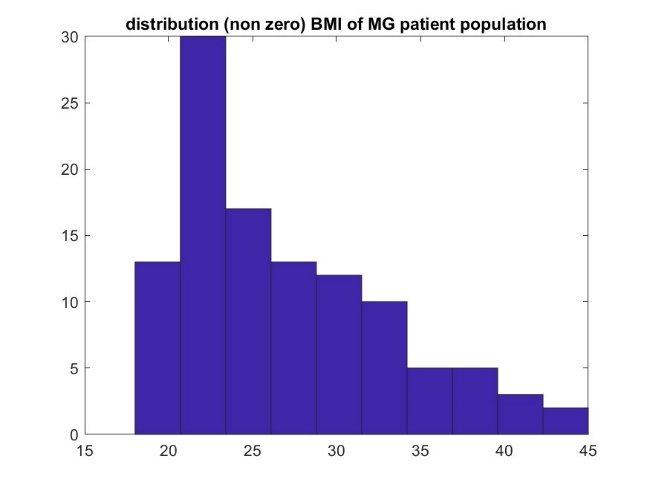


**Supplementary figure 1:** Properties of the MGTX patient population with respect to Age (Panel A) and BMI (Panel B).


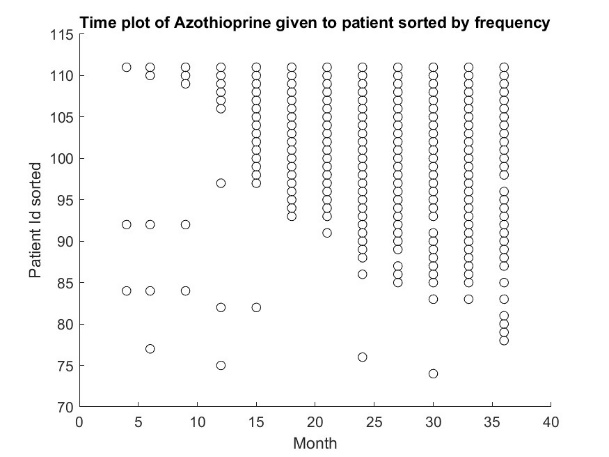

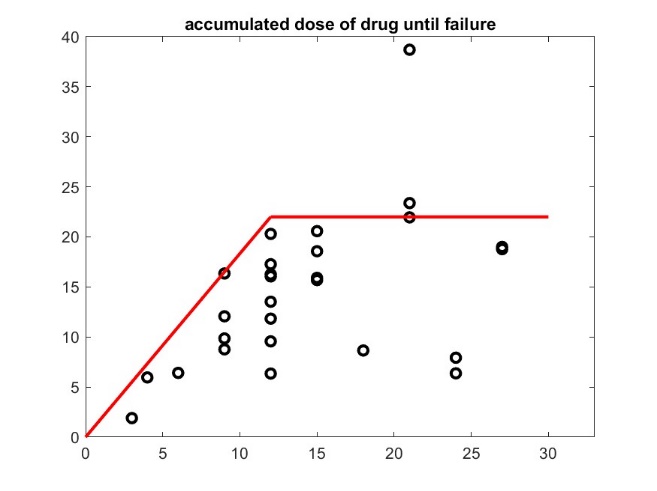


**Supplementary figure 2:** Temporal behavior of dose of azothiopine given to patient during the MGTX trial: once a patient is on this drug, he usually stays on it. Failure being define as the time patient starts to be on azothiopine, it seems that there is a saturation effect of the accumulated dose of prednisone that corresponds to the time of failure of the treatment.


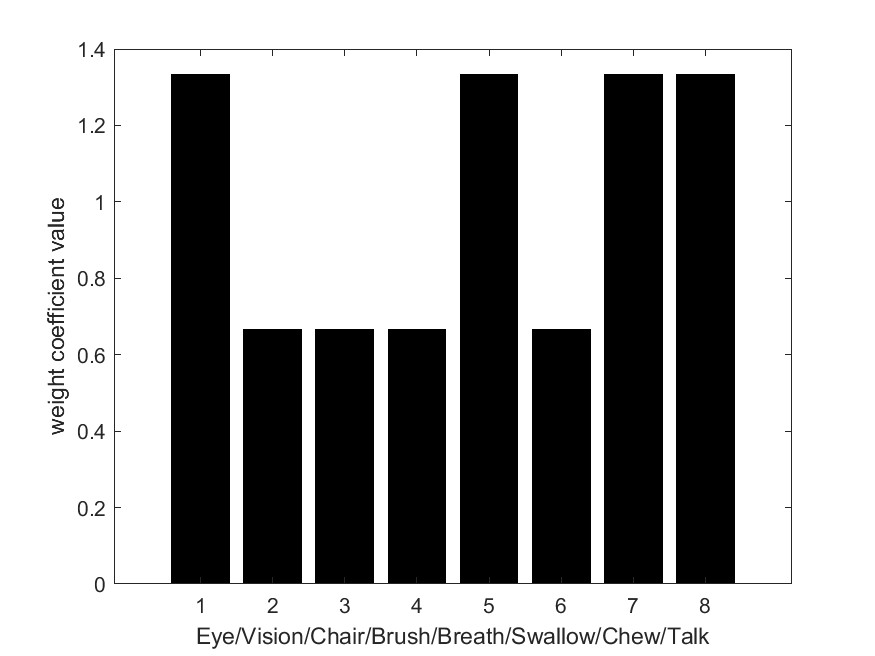


**Supplementary figure 3:** Optimum weight function for the composite ADL score.


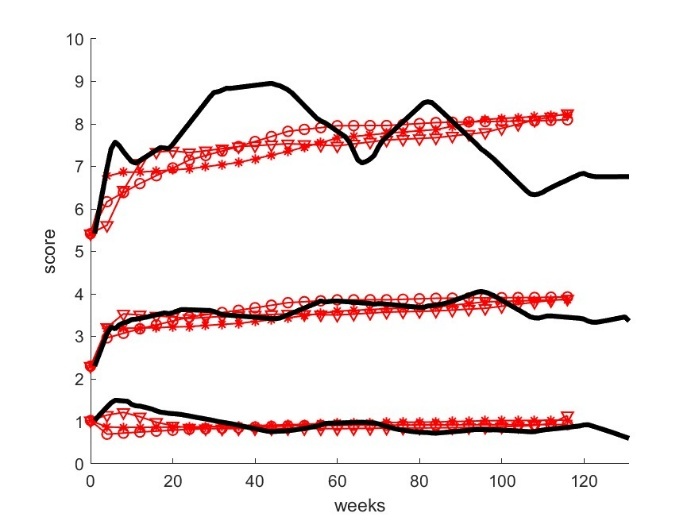

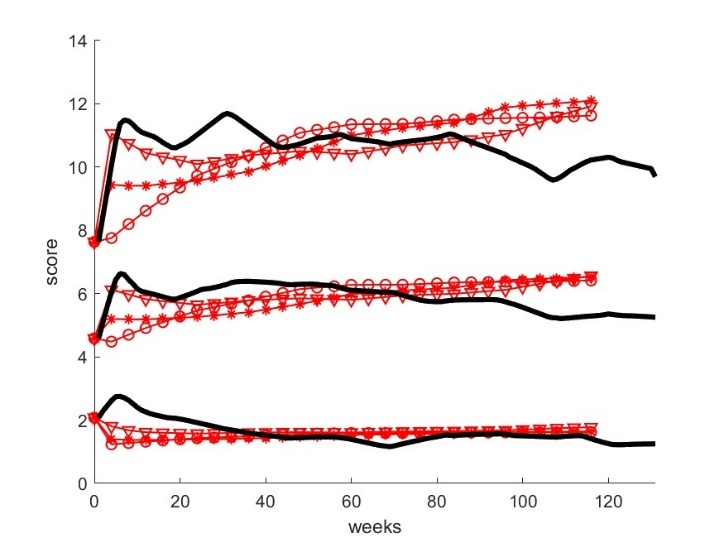


**Supplementary figure 4:** Model fitting for all three cluster scores curves. O, *, v are respectively for drug cluster input 1 to 3


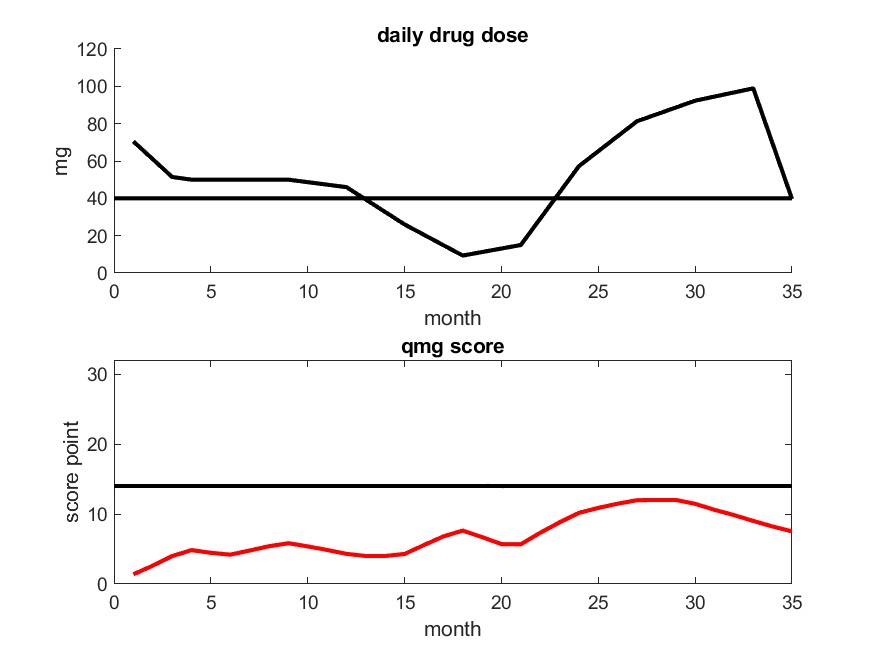

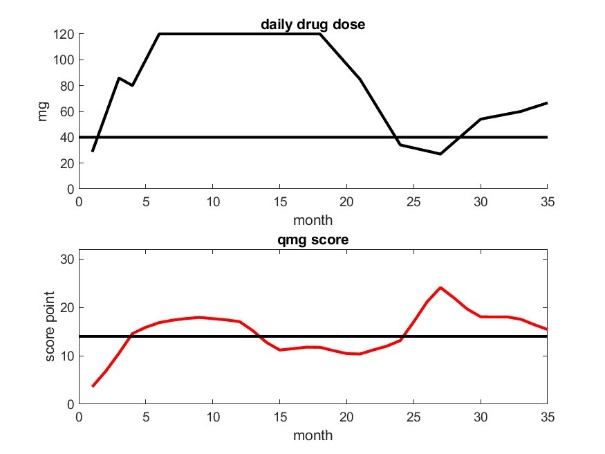


Figure A5 .1 Figure A5.2


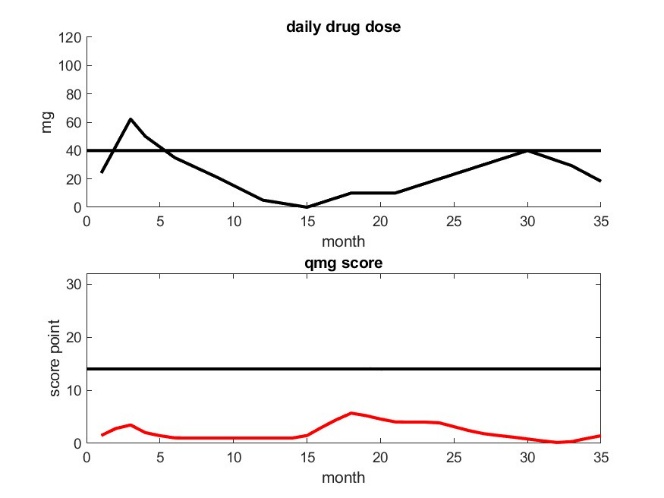

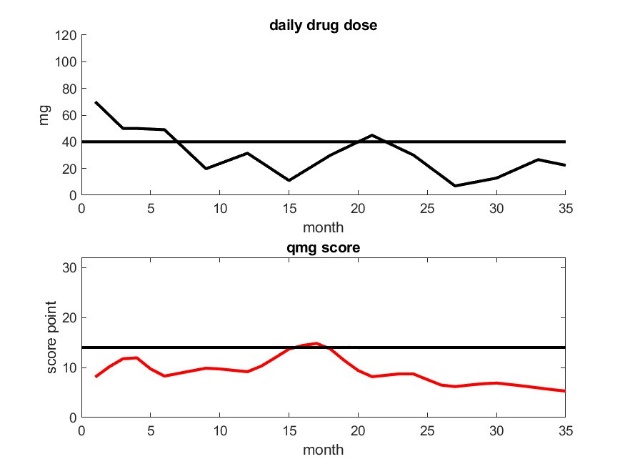


Figure A5.3 Figure A5.4

**Supplementary figure 5**: Model fitting for all three cluster scores curves. O, *, v are respectively for drug cluster input 1 to 3.


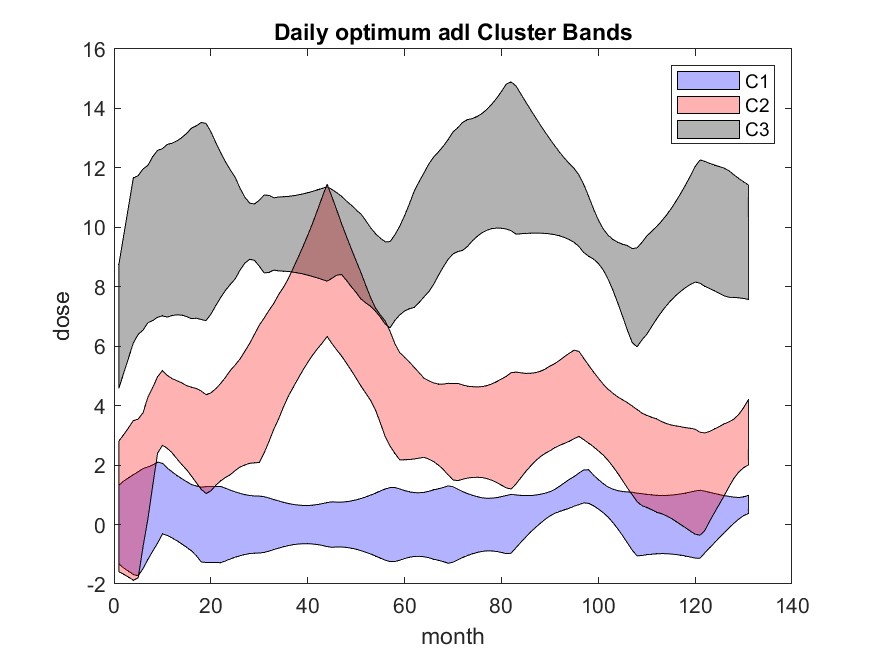


**Supplementary figure 6:** result of the clustering of the ADL modified score for patient trajectory, using a weight combination that corresponds to an improved prediction index.

| Si \ Yj | 1 | 2 | 3 |
| --- | --- | --- | --- |
| 1 | (2.1 , 0.96) | (0.5 , 4.1) | (0.25 , 8.4) |
| 2 | (2 , 1.2 ) | (0.5 , 4.6) | (0.25 , 9.6) |
| 3 | (2.4 , 1.2) | (0.5 , 5.3) | (0.25 , 11) |

**Supplementary table 1**: best "growth rate" α and the "carrying capacity" $S_{capacity}$ to match the score curves corresponding to each pair of cluster intersections (Si,Yj), i=1..3, j=1..3.
